# Neglected tropical diseases in Republic of Guinea: disease endemicity, case burden and the road towards the 2030 target

**DOI:** 10.1093/inthealth/ihad036

**Published:** 2023-05-26

**Authors:** Mahamoud Sama Cherif, Mory Keita, Prabin Dahal, Timothé Guilavogui, Abdoul Habib Beavogui, Lamine Diassy, Mohamed Conde, Abdoulaye Touré, Alexandre Delamou

**Affiliations:** Faculty of Sciences and Health Technics, Gamal Abdel Nasser University of Conakry, Conakry, Guinea; Direction Regionale de la Santé de Faranah, Ministère de la santé et de l'hygiène publique, Faranah, Guinea; Service de Pediatrie, Hospital National Ignace Deen, Ministère de la santé et de l'Hygiène Publique, Conakry, Guinea; World Health Organization, Regional Office for the Eastern Mediterranean, Cairo, Egypt; Institute of Global Health, Faculty of Medicine, University of Geneva, Geneva, Switzerland; Centre for Tropical Medicine and Global Health, University of Oxford, Oxford, UK; Management and Programmes Coordination, Ministry of Health, Conakry, Guinea; Faculty of Sciences and Health Technics, Gamal Abdel Nasser University of Conakry, Conakry, Guinea; Centre National de Formation et Recherche en Sante Rurale de Maferinyah, Maferinyah, Guinea; World Health Organization, Guinea office, Landreah, Corniche Nord, Boîte postale 817, Conakry, Guinea; Service de Pediatrie, Hospital National Ignace Deen, Ministère de la santé et de l'Hygiène Publique, Conakry, Guinea; Faculty of Sciences and Health Technics, Gamal Abdel Nasser University of Conakry, Conakry, Guinea; Institut National de Santé Publique, Ministère de la Santé et de l'Hygiène Publique, Conakry, Guinea; Faculty of Sciences and Health Technics, Gamal Abdel Nasser University of Conakry, Conakry, Guinea; Centre National de Formation et Recherche en Sante Rurale de Maferinyah, Maferinyah, Guinea

**Keywords:** Guinea, neglected tropical diseases, 2021–2030 roadmap, NTDs

## Abstract

Neglected tropical diseases (NTDs) predominantly affect vulnerable and marginalized populations in tropical and subtropical areas and globally affect more than one billion people. In Guinea, the burden of NTDs is estimated to be >7.5 disability-adjusted life years per million inhabitants. Currently the Guinea NTDs master plan (2017–2020) has identified eight diseases as public health problems: onchocerciasis, lymphatic filariasis, trachoma, schistosomiasis and soil-transmitted helminthiasis, leprosy, human African trypanosomiasis and Buruli ulcer. In this review we discuss the past and the current case burden of the priority NTDs in Guinea, highlight the major milestones and discuss current and future areas of focus for achieving the 2030 target outlined by the World Health Organization.

## Introduction

Neglected tropical diseases (NTDs) predominantly affect vulnerable and marginalized populations in tropical and subtropical areas and globally affect more than one billion people.^1^ To prevent and control NTDs, the World Health Organization (WHO) launched its first road map in 2012.^2^ Substantial gains made in the last decade include 500 million fewer people requiring interventions against NTDs in 2020 compared with 2010 and 40 countries, territories and areas have eliminated at least one NTD.^1^ The 2020 WHO road map built on the achievements of the first WHO road map has outlined several targets to be achieved by the year 2030.^1^ The overarching target is to achieve a 90% reduction in the number of people requiring interventions against NTDs, for at least 100 countries to eliminate at least one NTD, to eradicate two diseases (yaws and guinea worm) and to achieve a 75% reduction in the disability-adjusted life years (DALYs) related to NTDs.^1^

In Guinea, the National Programme for the Control of NTDs was launched in 2018 and drafted the Guinea NTDs master plan (2017–2020) to combat eight NTDs that were considered a public health problem: onchocerciasis, lymphatic filariasis (LF), trachoma, schistosomiasis and soil-transmitted helminths (STHs), leprosy, human African trypanosomiasis (HAT) and Buruli ulcer (BU).^3^ In addition to the recognisable burden of malaria and high neonatal mortality,^4–6^ Guinea also bears a substantial burden of NTDs, with an estimated 7.5 DALYs per million inhabitants attributed to NTDs.^1,7^

In this review we discuss the past and the present situation of the eight NTDs identified by the Guinean national control program along with other emerging or existing infections, highlight major milestones and discuss current and future areas of focus for achieving the 2030 target.

## Priority NTDs in Guinea: burden and progress

### Onchocerciasis (river blindness)

Onchocerciasis is a major infectious cause of blindness in the world. The Onchocerciasis Control Programme in West Africa (OCP), launched in 1974, has led to a steady decline in the global burden of the disease. The OCP initially began in seven countries (Benin, Burkina Faso, Cote d'Ivoire, Ghana, Mali, Niger and Togo) with the objective of eliminating onchocerciasis as a disease of public health concern. Prior to Guinea joining the OCP in 1986, the transmission was hyperendemic, with a very high disease prevalence (>60% prevalence in Upper Guinea in the 1980s and 20 000 blind people).^3,8^ A large-scale survey carried out in late 1980s as part of the OCP estimated a high burden of onchocerciasis; 14 villages in Guinea were identified as hyperendemic, with an overall disease prevalence of 65% and blindness prevalence of 6.5%.^9,10^ In order to reduce the burden of the disease, implementation of the OCP in Guinea began in 1987 and the program ran until 2002 in two distinct phases. During the first phase (1987–1997), larviciding was carried out and ivermectin treatment was administered by a mobile team of health workers. This combined use of vector control and ivermectin led to a rapid interruption of transmission.^11^ In the second phase (1997–2002), the control activities focused on larviciding under community-directed treatment with ivermectin (CDTI). From 2002 onwards, control activities have been implemented under CDTI.

The success of the OCP has led to a reduction in the prevalence of onchocerciasis in Guinea from 43–88% during the period of hyperendemic transmission to 0–15% in 2002.^3^ From 2003, the fight against onchocerciasis and other causes of blindness, including trachoma, were merged into a single program called the National Onchocerciasis and Blindness Control Program (PNLOC). To sustain the control activities, 2 570 255 Guineans received ivermectin treatment in 2009. Although the disease was considered endemic in Guinea until 2015,^12^ onchocerciasis is now no longer a major public health concern in the country. A recent modelling study has indicated a high environmental suitability index for disease transmission in most regions of Guinea expect the western coastal regions.^13^ Guinea has just completed mass drug administration (MDA) with ivermectin in 23 of 38 districts, with the last round completed in September 2022. The overall target is to achieve elimination by 2030.

### LF (elephantiasis)

In Guinea, the LF elimination program was initiated in 2000 after the WHO launched its global elimination program.^14^ A survey carried out across 46 sites in 2005 found a median disease prevalence of 0.5% (range 0.0–23.0).^15^ In 2013 the NTDs master plan reported a prevalence of circulating filarial antigens of *Wuchereria bancrofti* to be between 4.5 and 46.3% and a microfilariae prevalence of 3.0–6.7%.^16^ There has been a gradual reduction in the prevalence of LF in the past 2 decades: the estimated prevalence was 2–5% during 2000–2010 and <2% during the period 2010–2018.^17^ In 2013, 0.5% of the total population required preventative chemotherapy.^18^ MDA activities have been highly successful, with achievement of 100% geographic coverage in 2020.^19^ Resistance to permethrin and deltamethrin has been identified in Faranah and can be a challenge for the vector control activities.^20^ Currently a transmission assessment survey (TAS) is being undertaken. In 2022, the first assessment (pre-TAS) survey was conducted in 10 districts, with an overall prevalence observed of 0.0–0.43%. During the NTDs annual review workshop, which took place on 26–28 December 2022, it was reported that 11 districts underwent pre-stop surveys (TAS1), with an overall prevalence estimated at 0–5.4% (personal communication, Michel Sagno).

### Blinding trachoma

Guinea was one of the five countries that accounted for nearly half of the global burden of active trachoma.^21^ In 2011, the disease prevalence among children <10 y of age was 33% in a large nationwide survey.^3^ Most of the surveys conducted during the 2010–2019 period indicated an overall disease prevalence of <5% in most of the survey locations, expect in Faranah and Dinguiraye, which had a high prevalence of 30.0–49.9% in 2011.^22^ The country has implemented several components of the WHO-supported SAFE (surgery for treatment of advanced cases, antibiotics for follicular stage, facial cleanliness and environmental improvement) strategy since 2004. MDA with azithromycin and tetracycline ophthalmic ointment (preventive chemotherapy) began in 2013 and covered the nine health districts of the Upper Guinea region where the prevalence of follicular trachoma is >10%. A target date of 2015 was set for elimination of blinding trachoma.^2^ However, trachoma is still endemic in 31 of the 38 health districts and 3.6 million people required treatment in 2018.^23^ National trachoma mapping surveys (27 surveys) were conducted during 2011–2016, which reported an adjusted prevalence of trachomatous inflammation-follicular (TF) that ranged from 1.0 to 41.8%, while the adjusted point prevalence of trachomatous trichiasis (TT) ranged from 0.0 to 2.8% and an estimated 32 737 individuals required TT surgery.^24^ The fight against trachoma is a major priority for the national control program, especially in the Upper Guinea region and the northern part of Middle Guinea, where the disease prevalence remains high.

### Schistosomiasis (bilharzia)

Schistosomiasis has been a major public health problem in Guinea, with different surveys carried out between the 1930s and 1980s indicating a high prevalence of the disease (see Table 1). In Guinea, it is present in two major forms: the urinary disease caused by *Schistosoma haematobium* and the intestinal disease caused by *Schistosoma mansoni*. Transmission risk is high throughout the country, especially in the Upper Guinea region, where rice farming provides a suitable niche for the intermediate host (snail).^25^ In 1995, the prevalence of *S. mansoni* and *S. haematobium* among schoolchildren was 25% and 19.9%, respectively.^26^ In a survey carried out a decade later (2009–2010), the disease prevalence ranged from 23.5% to 49.6% for *S. haematobium* and 30.2% to 47.5% for *S. mansoni*.^3^ During 1996–2007, praziquantel, mebendazole, iron and folic acid donated by the WHO and the World Bank were the cornerstone of the schistosomiasis control program. In 2018, 4.03 million people required preventative chemotherapy, including 1.76 million school-age children.^27^ Treatment coverage of preventative chemotherapy remains suboptimal, with a national coverage of 33.6% in 2012, 31.2% in 2016, 42.7% in 2017 and 47.1% in 2018.^27^ Currently, all 38 districts are endemic for the disease.

**Table 1.** Eight NTDs listed in the Guinean NTDs program priority list

NTD	Endemicity	At risk/prevalence/case burden	Treatment coverage
Onchocerciasis (river blindness)	1990s: Macenta district in Forest region was meso- to hyperendemic in the late 1990s^98,99^ 2020: the disease is currently classified as being endemic^100^	Prevalence: overall national prevalence of 43–88% in 1980s and 0–15% in 2002^3^ An estimated 7 million people required preventive chemotherapy annually between 2017 and 2020^100^	81% in 2009 with 2 570 255 million ivermectin treatments^3^ 72% in 2017, 75% in 2018, 0% in 2019^23^
LF	Endemic as of 2020^19^	Prevalence: 2005: median prevalence of 0.5% (range 0–23.0)^15^ 2013: prevalence of circulating filarial antigens of *W. bancrofti* of 4.5–46.3% and microfilariae prevalence of 3.0–6.7%^16^ 2000–2018: estimated prevalence was 2–5% during 2000–2010 and <2% during 2010–2018^17^	Chemotherapy requirement: 2013: 0.5% of the total population required preventive chemotherapy^18^ Treatment coverage: 76% in 2017, 79% in 2018^23^ and 0% in 2019 among 7.48 million people needing treatment^23^ MDA coverage: MDA implemented with 100% geographical coverage as of 2020^19^
Trachoma	Endemic	Population at risk: 3 million (2007)^21^ 2011–2016: the population at risk of trachoma in the 18 health districts with a TF prevalence rate of ≥5% was estimated at 6 220 504 (USAID's report 2020) Prevalence: 2004: prevalence of active trachoma was found to be 5–23% in Kankan area, East Guinea^101^ 2011–2016: national trachoma mapping surveys (27 surveys)^24^ reported an adjusted prevalence of TF that ranged from 1.0% to 41.8% and adjusted point prevalence of TT ranged from 0.0% to 2.8%; an estimated 32 737 individuals required TT surgery 2012: studies from global trachoma mapping project found a TF prevalence of 4.3% among those ages 1–9 y and a TT prevalence of 0.3% among adults ≥15 y of age^102^	92% in 2017 to 35% in 2018^23^
Schistosomiasis	Endemic	Population at risk: population requiring preventative chemotherapy: 1.96 million (2010), 3.67 million (2015) and 4.03 million (2018)^27^ In 2019, 1.76 million school-age children required treatment^23^ Prevalence: 1939: prevalence of *S. haematobium* was 32–40% in Eastern Guinea and 30% in Western Guinea; *S. mansoni* prevalence was 68%^25^ 1955: 20% of the population infected with *S. haematobium* in 1955^25^ 1982: Eastern Guinea survey found *S. mansoni* prevalence of 57.9% in Guekedou, 48.0% in Macenta, 43.1% in Faranah, 41.8% in Nzerekore, 13.8% in Kerouane, 17.6% in Kankan, 17.7% in Dabola^25^ 1995: prevalence in schoolchildren: *S. mansoni* (25%), *S. haematobium* (19.9%)^3^ 1997: prevalence of 9.1% for urinary schistosomiasis among children across seven prefectures^103^ 2009–2010: 23.52% in Middle Guinea and 49.6% in Upper Guinea for urinary schistosomiasis; 30.22% in Middle Guinea and 47.5% in Upper Guinea for intestinal schistosomiasis^3^ 2010: Cross-sectional survey in Beyla and Macenta Prefectures, Forest Guinea found *S. haematobium* prevalence of 21% and *S. mansoni* prevalence of 66%^104^	National coverage of 33.6% in 2012, 31.2% in 2016, 42.7% in 2017 and 47.1% in 2018^27^ In 2019, 1.76 million school-age children required treatment but none received treatment^23^
STHs	All 38 districts are considered endemic^3^	1989: a survey carried out in Futa Djalon highlands found a helminths infection rate in Daka, Labe of 10.4%, 24.7% in Timbi Madina and 47.7% in Sombili^105^ 1997: prevalence of 60% for soil-transmitted nematodes among children across seven prefectures^103^ From 2000 onwards, among those <20 y of age, prevalence of hookworm (21.9% [95% CI 17.3 to 26.9]), roundworm (3.8 [95% CI 2.6 to 6.0]) and whipworm (1.7% [95% CI 1.0 to 3.9])^104^ 2010: cross-sectional survey in Beyla and Macenta Prefectures, Forest Guinea found hookworm prevalence of 51%; *Ascaris lumbricoides* prevalence of 8.1% and *Trichuris trichiura* prevalence of 2.4%^104^ In 2019, 2.38 million preschool and school-age children required treatment for intestinal worms but only 900 000 received treatment^23^	Not known In 2019, 2.38 million preschool and school-age children required treatment for intestinal worms but only 900 000 received treatment^23^
Leprosy	Endemic	Prevalence: 12 per 10 000 residents in 1990, 0.58 per 10 000 residents in 2008 and 0.21 per 10 000 residents in 2014 More than 150 cases have been reported annually since 2005^106^	Very rare
HAT	Endemic	Population at risk: 25.9% of the population is at risk (using 2011 census data), translating to 2.7 million people)^35^ Reported case burden: 4002 cases during 1932–1934, 19 334 cases in 1939, 85 659 cases during 1940–1953, 865 cases during 1990–2004^37^ 2005: prevalence of 0.9% (91/9637) in the mangrove areas^107^ 2007–2010: prevalence of 2.4 cases per 1000 inhabitant (31 cases out of 12 867 screened) in Forecariah district in coastal Guinea^108^ 2012–2016: 154 cases reported between 2012–2013 and 59 cases detected during the ebola outbreak in the coastal Guinea region^38^ For the past 3 decades, <150 cases are reported annually to the WHO data observatory	Not known
BU	Three of the 38 health districts are suspected endemic^3^	Notified cases: from 2002 to 2018, a total of 1582 cases were notified to the WHO^47^ Incidence: annual incidence of BU 0.83 cases per 100 000 population from 2011 to 2017 ^109^	No data available on antimicrobial coverage between 2011 and 2017^109^

**Table 2.** NTDs that are currently not on the priority list of the Guinean NTDs program

NTDs	Endemicity	Burden of the disease (case burden/mortality/DALYs)
Snakebite and scorpion envenoming	Endemic	2005: 6 million at risk; 100–150 envenomation per 100 000 inhabitants per year, with a lethality of 18% and 2% amputations^51^ 2018: 518 883 at risk of exposure to one or more venomous snake species and 427 253 at risk of exposure to any snakes for which no effective therapy exists^52^ 2019: 20% of the envenomings are due to elapids and the case fatality rate ranges from 15% to 27%^54^ Modelling study: annual deaths: 159 (95% CI 126 to 193)^55^, annual amputations: 192 (95% CI 118 to 327)^55^ , DALYs: 11 344 (8800–14 296)^55^ Data from Kindia Prefecture: 1980–1990: 584 snakebite cases with 2.2% case fatality rate^97^ 1997–1999: 379 cases reported with 18.2% fatality, 2.1% amputations^97^ 2002–2003: household survey reported 175 cases^110^ 2003–2004: 76 cases were treated at the Pasteur Institute, Kindia^111^ 2009–2010: 238 cases were treated at the Pasteur Institute, Kindia^61^ 2020: approximately 700 cases are reported annually^57^
Dengue	Not clear (under surveillance; epidemic prone disease)	2010: estimated mean apparent cases 192 067 and mean inapparent cases 593 001^18,66^ Sporadic case reports: a case of DENV was reported in 1981^62^, serologically detected dengue cases in a viral survey from the 1990s (described in Stoler et al. ^112^), four cases in 1996^63^ and one case in the Faranah regional hospital in 2006^64^
Chikungunya	Good consensus on the presence of CHIKV with a high environmental suitability for transmission^67^ (under surveillance; epidemic prone disease)	Case burden not known; only sporadic cases reported in the literature An outbreak occurred in 1992 (no details presented; see Table 2 of Powers and Logue^113^) A case of CHIKV reported in 1996^63^, 8 cases (4 confirmed, 4 suspected cases) reported in 2006^64^
Yaws	Previously endemic; current status is not clear (under surveillance; diagnosis is the problem, as the disease is very rare in Guinea)	32 900 cases identified in 1948^71^ A total of 1232 cases reported to the WHO regional office during 1974–1976^73^ 789 cases were reported to the WHO during 1977–1982^74^ In 2018, there were 3652 suspected cases (no reports of confirmed cases)^85^ In 2019, the disease was identified in a wild chimpanzee in the Sangaredi area^75^
Mycetoma	Endemic, as Guinea falls within the ‘mycetoma belt’^76^ (under surveillance)	Case burden not known; Only sporadic case reported in the literature 2010: two cases of mycetoma among two Guinean women resident in Cote d'Ivoire were reported (although the origin of infection is unclear)^77^ 2013: a case report of eumycetoma in a 29-year-old Guinean woman resident in France; the infection could have been acquired in Guinea through percutaneous contamination^78^
Taeniasis (*Taenia solium*)	Suspected endemic in 2018^114^ Inconsistent information on porcine cysticercosis (challenge with diagnosis)	Taenia *spp.* identified in 3.8% of the schoolchildren in a 1995 survey (species not described)^26^ Pig population of 104 000 reported to the WHO observatory in 2016 and 159 985 in 2020^115^
Leishmaniasis	Endemic for cutaneous leishmaniasis^116^ Non-endemic for Visceral Leishmaniasis with no autochthonous cases in 2018^116^	A case of cutaneous leishmaniasis reported in Kamsar in 1977^117^; no further data available
Scabies	–	Age-standardised 20–50 DALYs per 100 000 people^118^
Rabies	Endemic^68,82^	Mortality rate of more than three deaths per 100 000 population^82^
Guinea worm disease	Not endemic; certification of eradication achieved in 2009^80,81^	Eradicated

### STHs

All 38 districts are considered endemic for STHs.^3^ Among school-age children, the prevalence of hookworm (*Ancylostoma duodenale*), whipworm and roundworm was 43.9%, 13.9% and 9.5%, respectively, in 1995.^26^ From 2000 onwards, among those <20 y of age, the estimated combined prevalence of hookworm, whipworm and roundworm was 26.2% (95% confidence interval [CI] 21.9 to 31.4).^28^ Hookworm is the most prevalent helminth (21.9% [95% CI 17.3 to 26.9]) followed by roundworm (3.8 [95% CI 2.6 to 6.0]) and whipworm (1.7% [95% CI 1.0 to 3.9]). This translates to nearly 1.24 million cases of STHs every year. Annually, an estimated 1.4 million doses of anthelmintics are required for school-age children.^28^ Interrupting the transmission of STHs is considered highly unfeasible in Guinea and therefore STHs will continue to be a major NTD for the foreseeable future.^29^

### Leprosy

Leprosy, a disease caused by *Mycobacterium leprae*, predominantly affects the skin and the peripheral nerves and can lead to long-term disabilities. The disease is associated with stigma, especially when deformities are present. In 1988 the prevalence was 1.2% in Pita, a town in Fouta Djallon, Middle Guinea.^30^ In 1990 the prevalence of leprosy was nearly 12 cases per 10 000 inhabitants.^3^ A leprosy elimination campaign was organised in 1997 with the launch of several targeted programs. Overall, leprosy control has been based on screening of cases in all health centres, treatment of the identified cases, awareness and sensitisation campaigns and the establishment of reference centres for diagnosis and management of cases.^31^ This led to achieving the elimination target (<1 case per 10 000 residents) in all 38 health districts.^3^ The overall country prevalence was 0.58 cases per 10 000 inhabitants in 2008^31^ and 0.21 cases per 10 000 inhabitants in 2014.

Since 2002, the case detection system remains passive and in high-prevalence villages, leprosy days are organised. The disability percentage among incident cases remains high (9% in 2008).^3^ Recently, dapsone- and rifampicin-resistant leprosy has been identified;^32^ this is a major concern as treatment options for resistant cases are limited. Currently all the districts have reached the threshold of leprosy elimination (i.e. <1 case per 10 000 inhabitants).^31^ However, there are still hyperendemic areas with more than one case in some districts. A large proportion of the cases (86%) are multibacillary.^31^ The proportion of children among new cases was approximately 10% in 2008 and 7% in 2017;^31^ the proportion of mutilated among new cases was 17%, with an overall disease prevalence of 0.23 per 10 000 inhabitants in 2017. More than 9% of the patients screened have a degree 2 disability according to the WHO scale.^31^

### HAT (sleeping sickness)

HAT is caused by the parasite *Trypanosoma brucei*, which is transmitted by tsetse flies belonging to the genus *Glossina*. Two subspecies of *T. brucei* are pathogenic for humans: *T. b. gambiense* and *T. b. rhodesiense*. In West Africa, HAT is caused by the former species.^33^ The number of HAT cases decreased by 95% between 2000 and 2018 and the WHO has targeted to interrupt transmission of the disease (to zero cases) by 2030.^34^

Guinea has the largest number of HAT cases observed among West African countries. A total of 7.5% of the Guinean land area is considered at risk for *T. b. gambiense* and this translates to nearly 25% of the population living at risk.^35^ Transmission occurs mainly in the coastal regions with mangrove ecosystems (e.g. Boffa prefecture has the highest incidence in Guinea), as rice culture and attendance of pirogue jetties are associated with higher transmission risk.^33,36^ Annually there have been <150 cases reported to the WHO for the past 3 decades; the burden used to be much higher in the earlier half of the 20th century.^37^ The 2014–2015 Ebola virus disease (EVD) outbreak deeply impacted HAT control activities, with a direct impact on its surveillance and diagnosis; this led to an increase in the DALYs burden of at least 150.^38^ Subsequent intensification of activities has led to a decline in cases.^39^ The elimination target is likely to be achieved, as the National Sleeping Sickness Control Programme has been carrying out integrated vector control and medical intervention activities in collaboration with the Trypa-NO! partnership.^39,40^

Major challenges remain in the elimination of the disease from the regions with mangrove systems that harbour the main vector for transmission (*Glossina palpalis gambiensis*).^33,41^ Skin, a potential reservoir that has been previously unaccounted for, may need to be considered as a part of future control activities as the elimination goal is closer to being realised.^42^

### BU

BU is a chronic debilitating disease caused by *Mycobacterium ulcerans*. The exact mode of transmission to humans is unknown. Most of the cases are observed in children <15 y of age in West and Central Africa. The disease causes long-term disability in an estimated 25% of those affected.^43^ The history of BU in Guinea is relatively unknown, although its presence prior to 1980 has been reported in the literature, with further reported in 1995.^44^ The disease is currently endemic in Guinea and several areas of the country, especially the coastal regions in the western part, are predicted to be suitable for BU.^45,46^ From 2002 to 2018, a total of 1582 cases were reported to the WHO by the Guinean health services, with 102 cases reported in 2018,^47^ including 10 cases confirmed by polymerase chain reaction (PCR) In 2010.^31^ During 2014–2017, active screening and treatment of 348 cases were undertaken. The Guinean government has identified 3 of the 38 health districts as being suspected endemic.^3^

The control of BU has relied on early community-based detection and comprehensive care of patients to break the chain of transmission.^31^ As a part of the control program, doctors have been trained in case identification and management and a screening and treatment centre was established in N’Zérékoré in 2005. Guinea was one of the participating countries of the 2009 Cotonou Declaration.^48^ A recent study that classified any case of chronic cutaneous ulcer as BU reported a total of 389 cases in 2018, 400 cases in 2019 and 222 cases in 2020, with a wide geographic distribution of cases.^49^ The main target for 2030 outlined in the NTD road map 2021–2030 is to reduce the proportion of cases diagnosed in category III (severity grade) from 30% (baseline) to <10%.^43^ Diagnosis remains a major challenge, as samples for confirmatory PCR need to be sent to Cote d’Ivoire, leading to delayed case confirmation.^49^

## NTDs that are not identified as priority diseases

### Snakebite envenoming (SE)

The burden of SE prior to the 2000s remains unclear, with only a handful of annual deaths reported in the 1950s.^50^ In 2005 there were 6 million people at risk of SE and a total of 8000 cases were treated (Table 2).^51^ A global mapping study in 2018 estimated that 518 883 people were at risk of exposure to one or more venomous snake species and 427 253 were at risk of exposure to a snake for which no effective therapy exists.^52^ The following venomous snake species are found in Guinea: *Atheris chlorechis* (West African bush viper), *Bitis arietans* (puff adder), *Bitis nasicornis* (rhinoceros viper), *Bitis rhinoceros* (West African gaboon viper), *Dendroaspis polylepis* (black mamba), *Dendroaspis viridis* (western green mamba), *Dispholidus typus* (boomslang), *Echis jogeri* (Joger's carpet viper) and *Naja katiensis* (West African brown spitting cobra).^53^ A fifth of the envenomations are due to elapids and the case fatality rate ranges from 15% to 27%.^54^ Annually SE leads to 159 (95% CI 126 to 193) deaths and 192 (95% CI 118 to 327) amputations, with the DALYs burden estimated at 11 344 (95% CI 8800 to 14 296).^55^ The unavailability of antivenom and high treatment cost remains a major problem throughout the country, expect in Kindia—a region with one of the highest reported incidences of snakebites in the world.^51,56^ At the only dedicated snakebite clinic in Guinea, located in the Kindia region, approximately 700 cases are reported annually.^57^ However, the actual number of cases is likely to be underestimated, as many people may not seek treatment due to the potential financial burden.^57^

### Scorpion envenoming

The burden of scorpion envenoming remains unknown but is considered to be highly underreported; a case series described 75 cases during 2010–2012 in Conakry.^58^ Mortality is considered to be lower than that from SE.^59^

### Dengue

Currently there is a moderate level of evidence of the presence dengue virus (DENV) in Guinea^60^ and low–intermediate transmission risk.^61^ DENV serotype 2 was reported in 1981^62^ and in 1996,^63^ and a case was identified in Faranah in 2006.^64^*Aedes aegypti* mosquitoes, the primary vector for dengue transmission is present in Guinea.^65^ A modelling study in 2010 estimated a mean number of apparent cases of 192 067 (2.5th to 97.5th: 125 712 to 275 871); an apparent infection was defined as an infection with sufficient severity to modify a person's regular schedule.^18,66^ Apart from the sporadic case reports, there is relatively little information regarding the epidemiology of DENV in Guinea (Table 2).

### Chikungunya

There is good consensus on the presence of chikungunya virus (CHIKV) in Guinea, with high environmental suitability for transmission of the virus.^67^ A CHIKV outbreak occurred in 1992^68^ and eight confirmed/suspected cases of CHIKV were reported in 2006.^64^ There are no recent reports on CHIKV, although outbreaks were documented in neighbouring Senegal in 2015^69^ and Sierra Leone in 2012.^70^

### Yaws

Guinea was endemic for yaws in the 1940s, with a total of 32 900 cases identified in 1948.^71^ Mass treatment campaigns in the 1950s and 1960s led to a global decline in the burden of yaws by 95%.^72^ However, there was a resurgence in Africa after curtailment of control activities in the 1970s and 1980s. A total of 1232 cases were reported to the WHO during 1974–1976,^73^ with a further 789 cases reported in 1977–1982.^74^ In 2019, the disease was identified in a wild chimpanzee in the Sangaredi area.^75^ While the disease was previously endemic in the country, the current status remains unknown.

### Mycetoma

Despite Guinea falling within the ‘mycetoma belt’,^76^ the burden of the disease remains unknown. Description of the disease is limited to case reports among citizens living aboard. In 2010, two cases of mycetoma were reported in Guinean females.^77^ Another case report described eumycetoma caused by *Exophiala jeanselmei* in a 29-year old Guinean woman (resident in France for the last 10 y)—the infection could have been acquired in Guinea through percutaneous contamination.^78^

### Taeniasis

*Taenia solium* is suspected to be endemic in 2018, with a pig population of 104 000, including the practice of backyard pig production.^79^ Infection with *T. solium* (pork tapeworm) can cause the intestinal infection taeniasis, which has no major impact on human health. Ingestion of *T. solium* eggs via the faecal–oral route or by ingesting contaminated food or water causes infection in humans with the larval parasite in muscles, skin, eyes and the central nervous system (human cysticercosis). *Taenia* spp. were identified in 3.8% of the schoolchildren in a national survey in 1995 (species were not described).^26^

### Dracunculiasis (Guinea worm disease)

Certification of eradication of dracunculiasis was received in 2009.^80,81^

### Rabies

Guinea has one of the largest burdens of human rabies, with a mortality rate of more than three deaths per 100 000 population.^82^ Nearly 8000 incidents of animal bites were reported in Conakry during an 11-y study period (2002–2012).^83^

### Yellow fever

Outbreaks of yellow fever have been reported between 2006 and 2010, with a total of 80 cases identified, including 74 confirmed cases during an outbreak in 2005.^84^ Four suspected cases of yellow fever were reported from the Faranah region (in January–February 2022; immunoglobulin M negative eventually). There was one confirmed case identified in December 2022 (Faranah region); two suspected cases were reported in January 2023 (Faranah region; waiting for diagnostic confirmation) (personal communication, MSC). The disease is transmitted by *A. aegypti*, which is widely abundant in Guinea.

## Discussion

Guinea has implemented several programs to combat NTDs in the past 50 y. Major achievements during this period include certification of the eradication of dracunculiasis in 2009,^80,81^ the absence of confirmed reports of yaws in recent years^85^ and onchocerciasis is no longer considered a major public health concern.^12^ Eradication of yaws and dracunculiasis is one of the objectives of the WHO 2030 roadmap, and Guinea is well-positioned to achieve this. As part of a synergistic action and pooling of resources, the Ministry of Health decided in 2018 to group all NTDs into a single program called the National Program for the Fight Against NTDs. Some of the lessons learned from this program have been applied in the development of the revised NTDs master plan (2019–2023). A prior evaluation was carried out to review progress, identify common challenges and explore further opportunities for an integrated coordination of activities.^31^

Interrupting the transmission of STHs is considered highly unfeasible in Guinea and therefore STHs will continue to be a major NTD for the foreseeable future.^29^ Recently, dapsone- and rifampicin-resistant leprosy has been identified in a study among 24 patients.^32^ This is a major concern, as there are limited alternatives for the treatment of rifampicin-resistant cases. Despite the impressive control of trachoma, elimination of the disease from regions with mangrove systems currently remains a main challenge.^33,41^ Active screening and effective implementation of vector controls can help in achieving the zero transmission target by 2030.^86^

HAT is likely to meet the 2030 target of zero transmission; active screening of cases to tackle the human reservoir and effective implementation of tsetse fly vector control measures remain the key.^86,87^ The development of fexinidazole as an oral treatment for both the stages of HAT is an important development^88^; effective treatment compliance might be the key.^89^ The number of HAT cases is generally low and the burden appears to be mostly in the coastal region. Targeted active surveillance will lead to early identification of patients in stage 1, as passively diagnosed patients are more likely to present with later stages of the disease.^90^ The recent identification of skin as a potential reservoir needs to be considered as a part of future control activities for HAT.^42^

There are also further immediate challenges that threaten the different NTD programs: recurrent EVD outbreaks, the rampant use of counterfeit medicines and the ongoing coronavirus disease 2019 (COVID-19) pandemic. Unlike EVD outbreaks, which remain relatively localised, the ongoing COVID-19 pandemic has disrupted programs throughout the country. Guinea was one of the first countries in Africa to resume its MDA program during the COVID-19 pandemic without causing an observed increase in transmission.^91^ The resumption of activities coincided with the rainy season, and poor road conditions could have impeded the implementation progress.^92^

Sales of counterfeit medicines occur regularly despite the government's crackdown.^93,94^ According to government officials, >100 000 deaths per year are thought to be due to fake or falsified drugs.^95^ Despite a high burden attributed to counterfeit and substandard medicines, it remains largely neglected. Lessons learned from the cross-border collaboration (Mano River union) between Sierra Leone, Liberia, Guinea and Ivory Coast to combat onchocerciasis in West Africa can be useful.^96^ More of such initiatives are required to combat the NTDs in the region, as several diseases, such as HAT, have resurfaced after initial success in the 1960s,^37^ therefore sustained and coordinated control activities remain important.

Finally, only eight diseases are currently identified as major public health concerns in the Guinean NTDs master plan. Several diseases with a high burden currently remain underrecognised. For example, Guinea has a large burden of rabies, with mortality of more than three deaths per 100 000 population,^82^ and SE is clearly an underrecognised problem,^52^ especially in the Kindia region.^97^ The control program should consider including these NTDs as part of the national surveillance program.

## Conclusions

Guinea has made important progress towards combating NTDs, especially against yaws, dracunculiasis, onchocerciasis, trachoma and leprosy. Combatting NTDs through nationally coordinated efforts while dealing with the epidemic threats posed by viral illnesses should be a national priority.

## Data Availability

All the data used are available within the article.
